# Invasive mechanical ventilation as a risk factor for acute kidney injury in the critically ill: a systematic review and meta-analysis

**DOI:** 10.1186/cc12743

**Published:** 2013-05-27

**Authors:** Johannes PC van den Akker, Mahamud Egal, Johan AB Groeneveld

**Affiliations:** 1 Department of Intensive Care, Erasmus Medical Centre, 's-Gravendijkwal 230, 3015 CE, Rotterdam, The Netherlands

**Keywords:** Mechanical ventilation, acute kidney injury, meta-analysis, epidemiology, risk factors

## Abstract

**Introduction:**

Mechanical ventilation (MV) is commonly regarded as a risk factor for acute kidney injury (AKI) in the critically ill. We investigated the strength of this association and whether settings of tidal volume (Vt) and positive end-expiratory pressure (PEEP) affect the risk for AKI.

**Methods:**

We performed a systematic review and meta-analysis using studies found by searching MEDLINE, EMBASE, and references in relevant reviews and articles. We included studies reporting on a relation between the use of invasive MV and subsequent onset of AKI, or comparing higher with lower Vt or PEEP and subsequent onset of AKI. All studies clearly stating that MV was initiated after onset of AKI were excluded. We extracted the proportion with and without MV and AKI. We included 31 studies on invasive MV.

**Results:**

The pooled odds ratio (OR) for the overall effect of MV on AKI was 3.16 (95% CI 2.32 to 4.28, *P *<0.001). Nearly all subgroups showed that MV increases the risk for AKI. The pooled OR for studies with a multivariate analysis including MV as a risk factor for AKI was 3.58 (95% CI 1.85 to 6.92; *P *<0.001). Different settings of Vt and PEEP showed no effect.

**Conclusions:**

Invasive MV is associated with a threefold increase in the odds of developing AKI and various Vt or PEEP settings do not modify this risk. The latter argues in favour of a haemodynamic origin of AKI during MV.

## Introduction

Acute kidney injury (AKI) is - depending on the definition used - a common complication in the intensive care unit (ICU) with a high mortality, while it may also adversely affect long-term survival [1]. It affects up to 29% of patients who are mechanically ventilated [2]. In 1947, Drury *et al. *[3] were the first to describe the effects of positive airway pressure on renal function in healthy individuals. Since then, studies have demonstrated that mechanical ventilation (MV) affects the kidney [4]. However, a causal or epidemiological relation between MV and AKI has only been suggested in narrative reviews [4-6]. Kuiper *et al. *[4] proposed that MV may lead to the development of AKI through haemodynamic factors or ventilator-induced lung injury by triggering a pulmonary inflammatory reaction and subsequent systemic release of inflammatory mediators. Some studies specifically examined the release of these mediators during MV [7-9]. The precise relation between MV and subsequent AKI remains unclear, however.

In this systematic review, our primary objective was to answer the following questions: does invasive MV contribute to the development of AKI in critically ill adult patients, and could differences in ventilator settings like tidal volume (Vt) and positive end-expiratory pressure (PEEP) have an effect on the development of AKI? A secondary objective was to answer the question whether there is a difference between invasive MV and non-invasive MV (NIV) in the risk for AKI.

## Materials and methods

### Study eligibility and criteria

We performed a systematic literature search to identify studies reporting on the proportion of invasive MV used in critically ill adult patients that did or did not develop AKI, or reporting on the occurrence of AKI in patients ventilated with different ventilation strategies, like different Vt or levels of PEEP. We excluded studies clearly reporting that invasive MV was initiated after the onset of AKI and studies in which renal function was evaluated during a mean time interval shorter than 48 hours. Animal studies, articles not in English, studies unavailable as full text, and studies of paediatric patients were excluded.

We searched the MEDLINE (1966 to present) database via PubMed and the EMBASE (1980 to present) database (last search January 2012) using the following keywords: 'mechanical ventilation', 'acute renal failure', 'acute kidney injury', 'critically ill', 'risk factor', 'predictor', 'PEEP', 'tidal volume', and 'ventilation strategy'; see Table S1 in Additional file 1 for an example of a search strategy. In order to find studies not primarily reporting on the relation between invasive MV and AKI, the Medical Subject Heading label in PubMed was not applied. Instead, we used the text word [tw] label. We used the 'related articles' function in PubMed to identify eligible studies that were not found by the main search queries. References of those studies considered for inclusion and the references of the review articles were hand searched for eligibility. The 'cited reference search' function of the Web of Knowledge (Thomson Reuters) was also used to find potential studies citing those studies considered for inclusion.

Two authors (JPCvdA and ME) screened the title and abstract of the studies considered for inclusion, and in case of doubt both screened the full-text article. Two authors (ME and JPCvdA) then assessed the full-text article for eligibility and inclusion into the meta-analysis or the review in an unblinded manner. Disagreement was settled either by consensus or the third author (ABJG) made the definitive decision.

Using a predefined study form, one author (ME) extracted the following variables: study characteristics; patient characteristics; inclusion and exclusion criteria; definition of AKI used; time until MV was initiated; definition and settings of ventilation strategies used; time until onset of AKI; total study population; group sizes; number of patients in each group receiving invasive MV or developing AKI; and, if available, odds ratios (OR) - with confidence intervals (CI) or with *P *values - for invasive MV as a risk factor in univariate analysis, multivariate analysis or both. A second author (JPCvdA) checked all the extracted data. For patients in a postoperative setting, we categorised those receiving MV for 24 hours or less as not receiving MV. We used the term AKI for all forms of renal failure or dysfunction in the selected studies. We categorised the studies according to inclusion diagnosis and whether acute lung injury/acute respiratory distress syndrome (ALI/ARDS) was present or not. Also, we evaluated whether MV clearly preceded the onset of AKI.

We performed five analyses to answer the questions of the primary objective: one to answer the question whether MV has an effect on the onset of AKI overall and in subgroups according to inclusion diagnosis; one on the same question according to the subgroups whether ALI/ARDS was present or not; one on the same question but only using the pooled results of studies specifically analysing MV as a risk factor for AKI in multivariate analysis; one on studies with higher and lower Vt; and one on studies with higher and lower PEEP. Two separate analyses were performed to examine the difference between studies in which MV clearly preceded the diagnosis of AKI and in which this temporal relation was not completely clear. To answer the secondary objective we analysed studies comparing invasive MV with NIV.

### Statistical analysis

We calculated the OR and corresponding 95% CI using the total number of patients with and without AKI, and the proportion receiving invasive MV. The same was done with the studies examining different ventilator settings. If these data were not available, we used the reported OR and 95% CI. The reported univariate and multivariate OR and 95% CI were examined for rounding errors, and adjusted accordingly. The OR and corresponding 95% CI were put into a meta-analysis using a generic inverse variance method and a random effects model due to expected heterogeneity between studies. Heterogeneity was analysed using the I^2 ^statistics, and the thresholds for interpretation were used as defined in the Cochrane Handbook [10]. Publication bias was assessed visually using funnel plots. Review Manager (RevMan version 5.1. Copenhagen: The Nordic Cochrane Centre, The Cochrane Collaboration, 2011) was used for the analysis and creating the forest and funnel plots.

## Results

### Search

**Figure 1:**
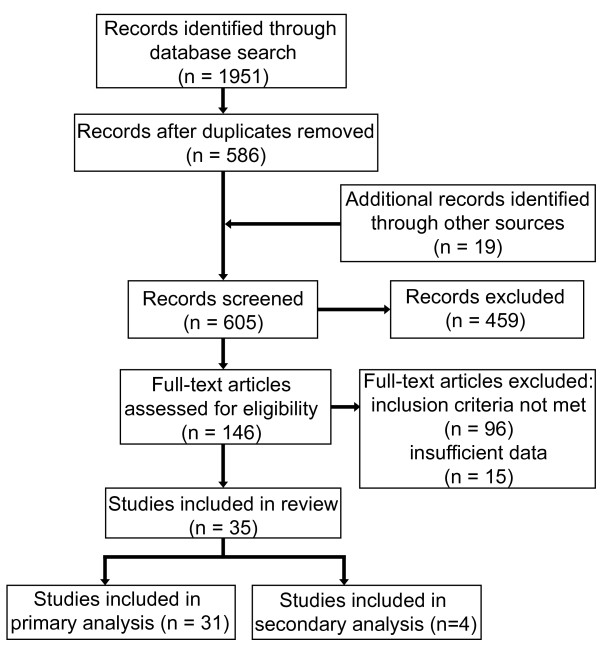
**Flow chart of search results and study selection**.

Our search strategies resulted in a total number of 1,951 citations (Figure 1). We also identified 19 studies through other sources. After removing duplicates, 605 remained. Of these, 459 met our exclusion criteria after screening the titles and abstracts. We subsequently assessed the remaining 146 full-text articles and 111 were excluded because inclusion criteria were not met or due to insufficient data. Of the remaining 35 studies, we included 31 studies in our primary analysis. The study characteristics of 23 studies [11-33] reporting on the use of invasive MV and renal function in a total number of 10,333 patients are presented in Table 1 and Table S2 in Additional file 1. Of these, seven studies [11,12,16,19,23,27,31] specifically analysed MV as a risk factor for AKI in multivariate analysis. The ventilator settings - Vt and PEEP - and study characteristics of nine studies [11,34-41] with a total number of 1,800 patients are presented in Table 2 and 3 and Table S2 and S3 in Additional file 1, respectively. The remaining four studies [2,42-44] comparing invasive MV with NIV were included in our secondary analysis.

**Table 1:** Summary of the characteristics of the studies comparing patients with and without mechanical ventilation.

Author/year	Inclusion diagnosis	Subgroup	Criteria type used	MV precedes AKI
Mataloun 2006 [17]	General population	Mixed	Absolute sCr rise	Yes

Payen 2008 [18]	General population	Mixed	Absolute sCr rise	Unclear

Fonseca Ruiz 2011 [26]	General population	Mixed	AKIN	Unclear

Medve 2011 [30]	General population	Mixed	AKIN	Unclear

Piccinni 2011 [33]	General population	Mixed	RIFLE	Unclear

				

Brito 2009 [19]	CABG	Cardiac disease	Dialysis, relative and absolute sCr rise	Yes

Marenzi 2010 [24]	STEMI with cardiogenic shock	Cardiac disease	Relative sCr rise	Yes

				

Iglesias 2010 [22]	Orthotopic liver transplant	Gastro-intestinal	AKIN	Yes

Lopes 2011 [28]	Cirrhosis	Gastro-intestinal	RIFLE	Unclear

O'Riordan 2011 [31]	Paracetamol hepatotoxicity	Gastro-intestinal	AKIN	Unclear

				

Abdulkader 2010 [21]	2009 Influenza A (H1N1)	Influenza A	RIFLE	Unclear

Jung 2011 [27]	2009 Influenza A (H1N1)	Influenza A	RIFLE	Unclear

Martin-Loeches 2011 [29]	2009 Influenza A (H1N1)	Influenza A	AKIN	Unclear

Pettila 2011 [32]	2009 Influenza A (H1N1)	Influenza A	RIFLE	Unclear

				

Shortgen 2001 [12]	Severe sepsis or septic shock	Sepsis	Relative sCr rise	Yes

Yegenaga 2004 [15]	SIRS/sepsis	Sepsis	Absolute sCr rise	Yes

Hoste 2003 [14]	Surgical sepsis	Sepsis	Absolute sCr rise	Unclear

Lopes 2009 [20]	Sepsis	Sepsis	AKIN and RIFLE	Unclear

				

Vivino 1998 [11]	Trauma	Miscellaneous	Absolute and relative sCr rise	Yes

Rocha 2005 [16]	Lung transplant	Miscellaneous	RIFLE and dialysis	Yes

Lahoti 2010 [23]	AML or high-risk MDS	Miscellaneous	RIFLE	Yes

Létourneau 2002 [13]	Bone marrow transplant	Miscellaneous	Absolute rise and relative sCr rise	Unclear

Murugan 2010 [25]	Community-acquired pneumonia	Miscellaneous	RIFLE	Unclear

MV, mechanical ventilation; AKI, acute kidney injury; sCr, serum creatinine; CABG, coronary artery bypass grafting; STEMI, ST elevation myocardial infarction; SIRS, systemic inflammatory response syndrome; AML, acute myeloid leukaemia; MDS, myelodysplastic syndrome.

**Table 2:** Summary of the characteristics of the studies with different settings for tidal volume.

		Lower Vt	Lower Vt	Higher Vt	Higher Vt
**Author/year**	**Diagnosis**	**Vt (ml/kg)**	**PEEP (cm H2O)**	**Vt (ml/kg)**	**PEEP (cm H2O)**

Amato 1998 [34]	ARDS	6	16.4 ± 0.4	12	8.7 ± 0.4

Stewart 1998 [35]	ARDS	7.0 ± 0.7	8.6 ± 3.0	10.4 ± 1.4	7.2 ± 3.3

Ranieri 2000 [36]	ARDS	7,6 ± 1.1	14.8 ± 2.7	11.1 ± 1.9	6.5 ± 1.7

Parikh 2005 [37]	ALI/ARDS	6.2 ± 0.9	9.4 ± 3.6	11.8 ± 0.8	8.6 ± 3.6

Villar 2006 [38]	ARDS	7.3 ± 0.9	14.1 ± 2.8	10.2 ± 1.2	9.0 ± 2.7

Cortjens 2011 [41]	No ALI	6	7*^a^*	10	7*^a^*

Vt, tidal volume; PEEP, positive end-expiratory pressure; ARDS, acute respiratory distress syndrome; ALI, acute lung injury. Data presented as mean ± standard deviation. *^a^*Approximately.

**Table 3:** Summary of the characteristics of the studies with different settings for PEEP.

		Higher PEEP	Higher PEEP	Lower PEEP	Lower PEEP
**Author/year**	**Diagnosis**	**Vt (ml/kg)**	**PEEP (cm H2O)**	**Vt (ml/kg)**	**PEEP (cm H2O)**

Amato 1998 [34]	ARDS	6	16.4 ± 0.4	12	8.7 ± 0.4

Vivino 1998 [11]	Trauma	Not reported	>6	Not reported	<6

Ranieri 2000 [36]	ARDS	7,6 ± 1.1	14.8 ± 2.7	11.1 ± 1.9	6.5 ± 1.7

Parikh 2005 [37]	ALI/ARDS	6.2 ± 0.9	9.4 ± 3.6	11.8 ± 0.8	8.6 ± 3.6

Villar 2006 [38]	ARDS	7.3 ± 0.9	14.1 ± 2.8	10.2 ± 1.2	9.0 ± 2.7

Manzano 2008 [39]	No ALI	7.79 ± 1.71	5.78 ± 1.0	7.91 ± 1.40	0.12 ± 0.7

Meade 2008 [40]	ALI/ARDS	6.8 ± 1.4	15.6 ± 3.9	6.8 ± 1.3	10.1 ± 2.9

Vt, tidal volume; PEEP, positive end-expiratory pressure; ARDS, acute respiratory distress syndrome; ALI, acute lung injury. Data presented as mean ± standard deviation.

### Meta-analyses

**Figure 2:**
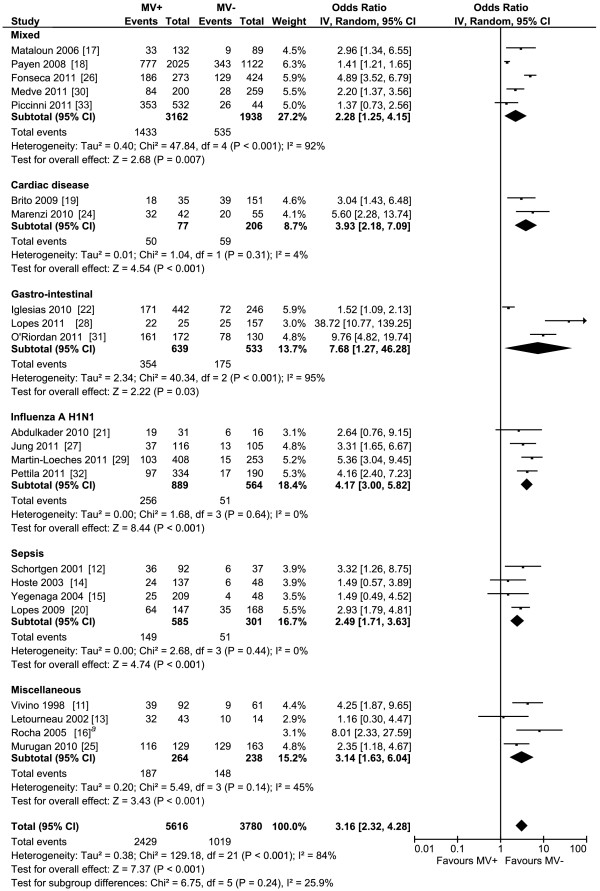
**Forest plot of the studies comparing the patients with and without mechanical ventilation on the onset of acute kidney injury categorised according to inclusion diagnosis**. MV+, with mechanical ventilation; MV-, without mechanical ventilation; IV, inverse variance; CI, confidence interval. *^a ^*Only OR reported.

**Figure 3:**
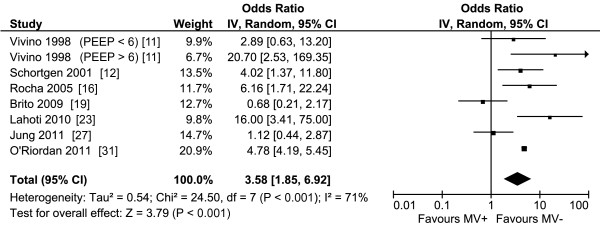
**Forest plot of the studies reporting on mechanical ventilation as a risk factor for acute kidney injury in multivariate analysis**. MV+, with mechanical ventilation; MV-, without mechanical ventilation; IV, inverse variance; CI, confidence interval; PEEP, positive end-expiratory pressure.

**Figure 4:**
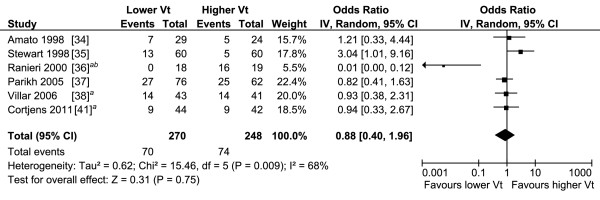
**Forest plot of the studies comparing higher with lower tidal volume on the occurrence of acute kidney injury**. IV, inverse variance; CI, confidence interval; Vt, tidal volume. *^a^*Acute kidney injury pre-randomisation omitted; *^b^*data extracted from bar graph.

**Figure 5:**
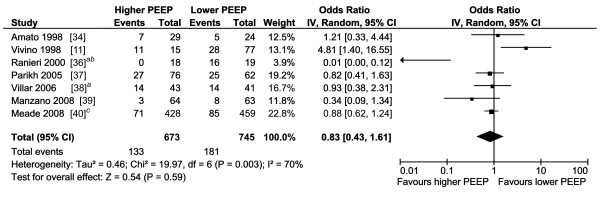
**Forest plot of the studies comparing higher with lower positive end-expiratory pressure on the occurrence of acute kidney injury**. IV, inverse variance, CI, confidence interval; PEEP, positive end-expiratory pressure. *^a^*Acute kidney injury pre-randomisation omitted; *^b^*data extracted from bar graph; *^c^*dialysis rates exclude patients receiving dialysis at the time of enrollment.

The pooled results of the 23 studies reporting on the use of invasive MV and renal function overall and of the subgroups are presented in Figure 2. Overall, the pooled OR for the occurrence of AKI when mechanically ventilated was about three. All subgroups showed that MV contributes to the development of AKI. The sepsis, influenza A H1N1, and cardiac subgroups show little heterogeneity, while the other subgroups have moderate to substantial heterogeneity. The pooled OR of the studies examining the studies in which MV clearly preceded the diagnosis of AKI and in which this temporal relation was not completely clear are also about three and are reported in Figure S5 and S6 in Additional file 1. The pooled results and the subgroups whether ALI/ARDS was present or not are reported in Figure S7 in Additional file 1, and also showed a pooled OR of about three. The subgroup analysis showed an OR of 2.4 and 5.6 whether ALI/ARDS was present or not, respectively. The pooled results of the studies reporting that MV is a risk factor for AKI using multivariate analysis are presented in Figure 3. The pooled OR for the occurrence of AKI in these studies was also about three and showed substantial heterogeneity. The variables adjusted for in the multivariate analysis are reported in Table S4 in Additional file 1. The pooled OR of studies with different settings for Vt and PEEP are reported in Figure 4 and 5. Different ventilator settings showed no effect on the development of AKI. A visual analysis of the funnel plots for each meta-analysis shows marked asymmetry for the studies reporting multivariate analyses and results of ventilation strategies (Figure S1 to S4 in Additional file 1).

The analysis comparing invasive MV with NIV on their respective contribution to the development of AKI is presented in Figure S8 in Additional file 1. AKI appears to develop more often in patients with invasive MV than in those with NIV.

## Discussion

In this systematic review and meta-analysis we show that invasive MV increases the odds for AKI by a factor of three, relatively independent of diagnostic subgroups. We also found an effect with the same magnitude after pooling studies specifically analysing MV as a risk factor for AKI in multivariate analysis. Neither Vt nor PEEP had an effect on the risk for AKI. AKI appears to develop more often in patients with invasive MV than in those with NIV. The latter observations argue in favour of haemodynamics as a major factor causing AKI during MV.

We realize that AKI is preceded by an insult and injury. It is possible that at the moment MV was started no failure was yet detected, but insult and injury had already occurred. Based on the published information, we therefore split the studies comparing MV with no MV into two groups. In the first group, invasive MV clearly preceded the onset of AKI. In the second group, no definitive distinction was possible between AKI at the start of MV or after invasive MV was initiated. The OR for the occurrence of AKI was about three in both groups. We observed that in four studies [20,26,29,31] an increase in AKI severity was associated with an increase in the percentage of patients who were mechanically ventilated, suggesting a causal relation. In these studies MV did not clearly precede the development of AKI. We can only speculate on the mechanisms of MV-attributable AKI, including haemodynamic factors and selective renal vasoconstriction by MV-induced sympathetic stimulation, among others. The results of the analysis of the subgroups in which ALI/ARDS was present or not argue against a biotrauma hypothesis of MV as a cause in the development of AKI.

Twenty-three of the selected studies are observational [11-33], which may result in confounding. To attenuate this risk, we used a random effects model, analysed diagnostic subgroups and performed a separate meta-analysis of studies analysing MV as a risk factor for AKI in multivariate analysis. In three studies [17,22,30], MV was a risk factor for AKI in univariate analysis, but not in multivariate analysis. This suggests that MV in these studies was not an independent risk factor. Also the substantial heterogeneity in the different meta-analyses suggests that MV is not the only risk factor for AKI. Arguably, the most important explanations of the heterogeneity are the different underlying aetiologies and differences in the severity of illness of the included patients, both leading to various *a priori *risks of AKI. The heterogeneity was low in some diagnostic subgroups - cardiac disease, influenza A H1N1_, _and sepsis - but high in others, suggesting a clinically significant association particularly in former conditions. Other explanations of the heterogeneity are a varying pre-admission renal function with different risks for AKI (Table S2 and S3 in Additional file 1) and the different criteria used to define AKI. With the recent introduction of the RIFLE and AKIN criteria, a lower threshold was set to define AKI compared with studies done before the introduction. This is especially true in the analysis of ventilator settings, in which studies were included with thresholds of AKI ranging from RIFLE-R to renal replacement therapy (Table S3 in Additional file 1).

Only one study in our analysis was specifically designed to examine the relation between different ventilator settings and the risk for AKI. This *post hoc *analysis by Cortjens *et al. *[41] reported no reduction in the development or worsening of AKI comparing low with high Vt MV, similar to the pooled result of the meta-analysis of Vt. Neither the meta-analyses of Vt nor PEEP showed evidence for an effect on the occurrence of AKI. The only study that showed a lower risk of AKI with higher PEEP and lower Vt was the one by Ranieri *et al. *[36]. The difference may be explained by other risk factors besides MV, for example a prolonged stay on the ICU: Ranieri *et al*. excluded all patients mechanically ventilated longer than eight hours before randomisation and had a follow-up of 96 hours after admission. The patients included in the other studies had already been mechanically ventilated for a longer time before inclusion or randomisation. They were followed until MV was stopped, 28 days after admission or until ICU discharge.

Two well-known studies examining ventilator settings and reporting on renal function [45,46] were not included in this meta-analysis - because the numerical values necessary to be analysed in this meta-analysis were not provided - but are important to discuss here. The ARDS Network trial [45] reported that patients in the low Vt group had more renal failure-free days as opposed to those in the high Vt group (20 ± 11 vs. 18 ± 11 days, *P *= 0.005). However, using a subset of the original ARDS Network trial and defining AKI by an increase of serum creatinine (sCr) 50% from baseline instead of the cutoff value of ≥2 mg/dl used in the original study, Parikh *et al*. showed that the incidence of AKI in both groups was similar [37]. The EXPRESS study [46] showed no difference between patients treated with low or high PEEP in terms of renal failure-free days when using a cutoff value of ≥3.4 mg/dl for sCr.

We found four studies comparing NIV with invasive MV [2,42-44]. When pooled together, AKI appears to develop more often in patients with invasive MV than in those with NIV (Figure S8 in Additional file 1). However, no evidence for such an effect is found when the studies are analysed separately in the diagnostic subgroups chronic obstructive pulmonary disease and mixed.

An estimate of the relative risk of developing AKI when mechanically ventilated using the data as presented in Figure 2 is 1.6. We calculated what this risk means for an ICU with 600 annual admissions, of which 500 receive MV. In a recent study, the risk of developing AKI in patients receiving MV was 29 percent [2]. In our example, 163 patients annually admitted will thus develop AKI. A hypothetical reduction of 20 percent in patients receiving invasive MV would lead to 11 patients who do not develop AKI annually. Given an ICU mortality of 50 percent, five patients will not die from MV-related AKI each year. We do not advocate reducing the appropriate use of MV, but we would like to emphasize that this study shows that besides well-known risk factors for AKI like hypotension, sepsis, intravenous contrast and antibiotics, MV is an additional one with clinical consequences.

This study has several limitations. The included studies were not specifically designed to answer prospectively the question whether MV has an effect on renal function and, therefore, a large diversity in studies, study designs, patient categories with different diagnoses, definitions of renal failure and severity of illness were combined. In some cases, it was not possible to extract from the published material definitively whether AKI was present at the start of MV or after MV was initiated.

## Conclusions

In conclusion, invasive MV is associated with a threefold increase in odds of AKI in critically ill patients. In general, Vt or PEEP settings do not seem to modify the risk.

## Key messages

• Invasive mechanical ventilation is associated with a threefold increase in odds of acute kidney injury in critically ill patients.

• Tidal volume or positive end-expiratory pressure settings do not seem to modify the risk.

• Acute kidney injury appears to develop more often in patients with invasive mechanical ventilation than in those with non-invasive ventilation.

## Abbreviations

AKI: acute kidney injury; ALI: acute lung injury; AML: acute myeloid leukaemia; ARDS: acute respiratory distress syndrome; CABG: coronary artery bypass grafting; CI: confidence interval; ICU: intensive care unit; IV: inverse variance; MDS: myelodysplastic syndrome; MV: mechanical ventilation; MV+: with mechanical ventilation; MV-: without mechanical ventilation; NIV: non-invasive ventilation; OR: odds ratio; PEEP: positive end-expiratory pressure; sCr: serum creatinine; SIRS: systemic inflammatory response syndrome; STEMI: ST elevation myocardial infarction; Vt: tidal volume.

## Competing interests

The authors declare they have no competing interests.

## Authors' contribution

JPCvdA and ABJG designed the study. JPCvdA and ME performed the search and the inclusion of studies, supervised by ABJG. ME performed the meta-analysis, under the supervision of JPCvdA and ABJG. JPCvdA and ME prepared the manuscript, and ABJG supervised and edited the manuscript. All authors read and approved the final manuscript.

## Supplementary Material

Additional file 1 **Table S1**. Search strategy for invasive mechanical ventilation as a risk factor in PubMed. **Table S2**. Characteristics of the studies comparing the patients with and without mechanical ventilation on the occurrence of acute kidney injury. **Table S3**. Characteristics of the studies examining different ventilator settings. **Table S4**. Variables adjusted for in multivariate analysis. **Figure S1**. Funnel plot of the studies comparing the patients with and without mechanical ventilation on the occurrence of acute kidney injury. **Figure S2**. Funnel plot of the studies reporting on mechanical ventilation as a risk factor for acute kidney injury in multivariate analysis. **Figure S3**. Funnel plot of the studies comparing low and high positive end-expiratory pressure on occurrence of acute kidney injury. **Figure S4**. Funnel plot of the studies comparing low and high tidal volume ventilation on occurrence of acute kidney injury. **Figure S5**. Forest plot of the studies in which mechanical ventilation clearly preceded acute kidney injury. **Figure S6**. Forest plot of the studies in which mechanical ventilation did not clearly precede acute kidney injury. **Figure S7**. Forest plot of the studies comparing the patients with and without mechanical ventilation categorised in subgroups whether ALI/ARDS was present or not. **Figure S8**. Forest plot comparing non-invasive with invasive mechanical ventilation on the occurrence of acute kidney injury. Click here for file
