# Supplementary material for: Invasive mechanical ventilation as a risk factor for acute kidney injury in the critically ill: a systematic review and meta-analysis
Source: Crit Care. 2013 May 27;17(3):R98. doi: 10.1186/cc12743 (PMC3706893; doi:10.1186/cc12743)
Supplement: Additional file 1 — Table S1. Search strategy for invasive mechanical ventilation as a risk factor in PubMed. Table S2. Characteristics of the studies comparing the patients with and without mechanical ventilation on the occurrence of acute kidney injury. Table S3. Characteristics of the studies examining different ventilator settings. Table S4. Variables adjusted for in multivariate analysis. Figure S1. Funnel plot of the studies comparing the patients with and without mechanical ventilation on the occurrence of acute kidney injury. Figure S2. Funnel plot of the studies reporting on mechanical ventilation as a risk factor for acute kidney injury in multivariate analysis. Figure S3. Funnel plot of the studies comparing low and high positive end-expiratory pressure on occurrence of acute kidney injury. Figure S4. Funnel plot of the studies comparing low and high tidal volume ventilation on occurrence of acute kidney injury. Figure S5. Forest plot of the studies in which mechanical ventilation clearly preceded acute kidney injury. Figure S6. Forest plot of the studies in which mechanical ventilation did not clearly precede acute kidney injury. Figure S7. Forest plot of the studies comparing the patients with and without mechanical ventilation categorised in subgroups whether ALI/ARDS was present or not. Figure S8. Forest plot comparing non-invasive with invasive mechanical ventilation on the occurrence of acute kidney injury. [file cc12743-S1.DOC]

# Additional file provided with this submission:

# Invasive mechanical ventilation as a risk factor for acute kidney injury in the critically ill: a systematic review and meta-analysis

Johannes P.C. van den Akker, Mahamud Egal, A.B. Johan Groeneveld

Department of Intensive Care Medicine, Erasmus Medical Centre, Mailbox 2040,

3000CA Rotterdam, The Netherlands

Corresponding Author: J.P.C. van den Akker (j.vandenakker@erasmusmc.nl)

**Legend**

**Table S1.** Search strategy for invasive mechanical ventilation as a risk factor in PubMed

**Table S2.** Characteristics of the studies comparing the patients with and without mechanical ventilation on the occurrence of acute kidney injury

**Table S3.** Characteristics of the studies examining different ventilator settings

**Table S4.** Variables adjusted for in multivariate analysis

**Figure S1.** Funnel plot of the studies comparing the patients with and without mechanical ventilation on the occurrence of acute kidney injury.

**Figure S2.** Funnel plot of the studies reporting on mechanical ventilation as a risk factor for acute kidney injury in multivariate analysis.

**Figure S3.** Funnel plot of the studies comparing low and high positive end-expiratory pressure on occurrence of acute kidney injury.

**Figure S4.** Funnel plot of the studies comparing low and high tidal volume ventilation on occurrence of acute kidney injury.

**Figure S5.** Forest plot of the studies in which mechanical ventilation clearly preceded acute kidney injury.

**Figure S6.** Forest plot of the studies in which mechanical ventilation did not clearly precede acute kidney injury.

**Figure S7.** Forest plot of the studies comparing the patients with and without mechanical ventilation categorised in subgroups whether ALI/ARDS was present or not.

**Figure S8.** Forest plot comparing non-invasive with invasive mechanical ventilation on the occurrence of acute kidney injury.

**Table S1.** Search strategy for invasive mechanical ventilation as a risk factor in PubMed

| 1. “intensive care”[tw] |
| --- |
| 1. “intensive care”[tiab] |
| 1. “critically ill”[tw] |
| 1. “critically ill”[tiab] |
| 1. 01 OR 02 OR 03 OR 04 |
| 1. “acute renal failure”[tw] |
| 1. “acute renal failure”[tiab] |
| 1. “acute kidney injury”[tw] |
| 1. “acute kidney injury”[tiab] |
| 1. 0 6 OR 07 OR 08 OR 09 |
| 1. “mechanical ventilation”[tw] |
| 1. “mechanical ventilation”[tiab] |
| 1. 11 OR 12 |
| 1. “risk factor”[tw] |
| 1. “risk factor”[tiab] |
| 1. “predictor”[tw] |
| 1. “predictor”[tiab] |
| 1. “mortality”[tw] |
| 1. “mortality”[tiab] |
| 1. 14 OR 15 OR 16 OR 17 OR 18 OR 19 |
| 1. 05 AND 10 AND 13 AND 20 |

**Table S2.** Characteristics of the studies comparing the patients with and without mechanical ventilation on the occurrence of acute kidney injury

| **Author/Year** | **n** | **Design** | **Population** | **Renal exclusion** | **Pre-admission sCr** | **Definition AKI** |
| --- | --- | --- | --- | --- | --- | --- |
| Vivino 1998 [11] | 153 | Prospective single centre | Trauma | None reported | None reported | SCr > 2 mg/dl or > 20% increase from baseline sCr when already > 2 mg/dl |
| Shortgen 2001 [12] | 129 | Prospective, multicentre  (n = 3) | Severe sepsis or septic shock | SCr > 320 μmol/l or dialysis | HES: 143 (88–203)  μmol/l  Gel: 114 (91–175); | two-fold increase in  sCr from inclusion or need for RRT |
| Letourneau 2002 [13] | 57 | Retrospective single centre | Bone marrow transplant | None of the patients had chronic renal failure at time of BMT | AKI+: 92.3 ± 24.1 μmol/l  AKI-: 86.3 ± 12.2; NS | Doubling of sCr or > 200 μmol/l |
| Hoste 2003 [14] | 185 | Retrospective single centre | Surgical sepsis | Pre-existing renal insufficiency ( sCr > 1.5mg/dl) | AKI+: 1.43 (1.05 to 2.4)  AKI-: 0.91 (0.71to 1.05); S | Rise from normal sCr (upper limit 1.0 mg/dl) to ≥ 2 mg/dl |
| Yegenaga 2004 [15] | 257 | Prospective single centre | SIRS/sepsis | SCr > 2mg/dl on the day patients developed SIRS/sepsis | AKI+: 1.3 ± 0.4 mg/dl  AKI-: 1.0 ± 0.3; S | sCr > 2 mg/dl or urine output < 400ml/24h |
| Rocha 2005 [16] | 296 | Retrospective single centre | Lung transplant | None reported | AKI+: 0.97 ± 0.23 mg/dl  AKI-: 0.88 ± 0.22; S | Dialysis |
| Mataloun 2006 [17] | 221 | Prospective single centre | Mixed | sCr > 1.5 mg/dl or renal transplant recipient | AKI+: 1.04 ± 0.29 mg/dl  AKI-: 0.88 ± 0.26; S | SCr > 1.5 mg/dl |
| Payen 2008 [18] | 3147 | Prospective multicentre  (n = 198) | Mixed | None reported | AKI+: 2.2 ± 2.2 mg/dl  AKI-: 1.0 ± 0.5; S | > 3.5 mg/dL (310 μmol/l) or a  urine output < 500 ml/day |
| Brito 2009 [19] | 186 | Prospective single centre | CABG | GFR <15 ml/min | 15.1% of all patients had sCr > 1.3 mg/dl | 50% increase in sCr if baseline > 1.3 mg/dl;  increase of 0.5 mg/dl if baseline < 1.3; or need for dialysis postoperatively during ICU stay |
| Lopes 2009 [20] | 315 | Retrospective single centre | Sepsis | Chronic kidney disease on dialyis or renal transplant patients | None reported | AKIN |
| Abdulkader 2010 [21] | 47 | Retrospective single centre | 2009 Influenza A (H1N1) infected | None reported | AKI+: 1.04 (0.70 to 1.75) mg/dl  AKI-: 0.95 (0.66 to 1.14); NS | RIFLE |
| Iglesias 2010 [22] | 688 | Retrospective multicentre  (n = 3) | orthotopic liver transplantation | Requiring RRT pre or perioperative | AKI+: 1.05 ± 0.40 mg/dl  AKI-: 1.05 ± 0.60; NS | Absolute increase of sCr ≥ 0.3 or increase of 50% from baseline. |
| Lahoti 2010 [23] | 537 | Retrospective single centre | AML or high-risk MDS undergoing induction chemotherapy | SCr > 1.5 mg/dl | 0.8-0.9 mg/dl | RIFLE |
| Marenzi 2010 [24] | 97 | Prospective single centre | STEMI complicated by cardiogenic shock at hospital admission, and treated by IABP and primary PCI | Patients in chronic peritoneal or hemodialytic treatment | AKI+: 1.3 (1.1 to 1.7)  AKI-: 1.1 (1.0 to 1.3) ; S | ≥ 25 % increase in sCr from baseline |
| Murugan 2010 [25] | 292 | Retrospective multicentre (n = 28) | Non-severe community acquired pneumonia | None reported | AKI+: 0.93 ± 0.3 mg/dl  AKI-: 0.89 ± 0.2; NS | RIFLE |
| Fonseca Ruiz 2011 [26] | 697 | Retrospective single centre | Mixed | Chronic renal support therapy (dialysis) before ICU admission | AKI+1: 0.8 ± 0.5 mg/dl  AKI+2: 0.8 ± 0.4 mg/dl  AKI+3: 1.08 ± 1.15 mg/dl  AKI-: 0.75 ± 0.4; NS | AKIN |
| Jung 2011 [27] | 221 | Retrospective multicentre  (n = 28) | 2009 Influenza A (H1N1) infected | End-stage renal disease on RRT | AKI+: 1.7 (1.1 to 2.7) mg/dl  AKI-: 0.9 (0.7 to 1.0); S | RIFLE |
| Lopes 2011 [28] | 182 | Retrospective single centre | cirrhosis | None reported | AKI+: 1.8 ± 1.2 mg/dl  AKI-: 0.9 ± 0.6 ; S | ≥ 50% increase in sCr from baseline or absolute increase of > 0.3 mg/dl |
| Martin-Loeches 2011 [29] | 661 | Prospective multicentre  (n = 148) | 2009 Influenza A (H1N1) infected | Chronic kidney disease receiving dialysis | None reported | AKIN |
| Medve 2011 [30] | 459 | Prospective multicentre  (n = 7) | Mixed | Chronic kidney disease on dialysis and renal transplant | AKI+: 117.2.5 (81 to 205) μmol/l  AKI-: 70 (57 to 87); S | AKIN |
| O'Riordan 2011 [31] | 302 | Retrospective single centre | Paracetamol hepatotoxicity | None reported | None reported | AKIN |
| Pettila 2011 [32] | 628 | Retrospective multicentre (n = 187) | 2009 Influenza A (H1N1) infected | None reported | None reported | RIFLE |
| Piccinni 2011 [33] | 576 | Prospective multicentre (n = 10) | Mixed | Endstage renal disease on chronic RRT | AKI+: 1.1 (0.8 to 1.6) mg/dl  AKI-: 0.8 (0.6 to 1.0); S | RIFLE (+ lowest urine output) |

MV, mechanical ventilation; AKI+, acute kidney injury present; AKI-, acute kidney injury not present; ARF, acute renal failure; HES, hydroxyethylstarch; Gel, gelatine; AML, acute myeloid leukemia; MDS, myelodysplastic syndrome; OLT, orthotopic liver transplantation; RRT, renal replacement therapy; CRRT, continuous renal replacement therapy; GFR, glomerular filtration rate; NS, not significant; S, significant; SIRS, systemic inflammatory response syndrome; CPR, cardiopulmonary resuscitation; STEMI, ST-elevation myocardial infarction; CABG, coronary artery bypass grafting; IABP, intra-aortic ballon pump; PCI, percutaneous coronary intervention; sCr, serum creatinine; BUN, blood urea nitrogen; ICU, intensive care unit; BMT, bone marrow transplant; LITU, liver intensive therapy unit.

**Table S3.** Characteristics of the studies examining different ventilator settings

| **Author/Year** | **n** | **Design** | **Population** | **Renal exclusion** | **Pre-admission sCr** | **Definition AKI** |
| --- | --- | --- | --- | --- | --- | --- |
| Amato 1998 [34] | 53 | RCT multicentre (n=2) | ARDS | None reported | None reported | - Dialysis |
| Stewart 1998 [35] | 120 | RCT multicentre (n=8) | ARDS | None reported | None reported | - Dialysis |
| Ranieri 2000 [36] | 44 | RCT multicentre (n=2) | ARDS | None reported | None reported | One or more:  - urine output ≤ 479 ml/24h or ≤159 ml/8h - serum BUN ≥ 100mg/dl - sCr ≥ 3.5mg/dl [310 µmol/l] |
| Parikh 2005 [37] | 132 | Case-control from a multicentre RCT | ALI/ARDS | sCr > 1.2 mg/dl | AKI+: 0.85 (0.5 to 1.1) AKI-: 0.90 (0.5 to 1.1); P = 0.3 | - ≥ 50% increase from baseline sCr |
| Villar 2006 [38] | 95 | RCT multicentre (n=8) | ARDS > 24hrs | None reported | None reported | - sCr > 2mg/dl [177 mmol/dl] - when pre-existing renal disease, doubling of admission sCr |
| Manzano 2008 [39] | 131 | RCT multicentre (n =3) | Non-hypoxemic without lung injury | None reported | None reported | - dialysis |
| Meade 2008 [40] | 887 | RCT multicentre (n = 30) | ALI/ARDS | Dialysis at time enrolment. | None reported | - dialysis |
| Cortjens 2011 [41] | 150 | RCT multicentre (n=2) | no ALI at onset of MV | None reported | None reported | - RIFLE - baseline sCr estimated using MDRD formula |

AKI, acute kidney injury; RCT, randomised controlled trial; ARDS, acute respiratory distress syndrome; BUN, blood urea nitrogen; sCr, serum creatinine; ALI, acute lung injury; MV, mechanical ventilation; MDRD, Modification of Diet in Renal Disease.

**Table S4.** Variables adjusted for in multivariate analysis

| **Study** | **Risk factors** |
| --- | --- |
| Vivino 1998 [11] | age in years, GCS (6-10 and ≤ 5), ISS (17-32 and >32), MAP (<80), rhabdomyolysis (Yes, CPK > 10000 and Yes, CPK < 10000), Shock (yes), hemoperitoneum (yes), long bone fracture (yes), abdominal trauma (yes) |
| Schortgen 2001 [12] | type of expander, fluid loading before study inclusion, use of vasoactive drugs |
| Rocha 2005 [16] | baseline GFR, diagnosis (other vs COPD), amphotericin-B (yes vs no) |
| Brito 2009 [19] | CPB (>115 min vs < 115 min), aortic clamping time (> 85 min vs < 85 min), stay in ICU over 3 days (yes vs no), need for IAB (yes vs no), hypotension (yes vs no), arrhythmia (yes vs no), use of inotropic drugs (yes vs no) |
| Lahoti 2010 [23] | age ≥ 55 years, male vs female, vasopressors, intravenous diuretics, vancomycin, amphotericin-B, WBC, albumin, non-fludarabine-based regimen |
| Jung 2011 [27] | Age (years), nosomical acquisition, no of co-morbidity, chronic kidney disease, hypertension, diabetes mellitus, immunosuppresed state, SOFA-score, APACHE-II score, shock, corticosteroid use |
| O'Riordan 2011 [31] | vasopressor use, admission phosphate, admission sodium, day 3 haematocrit, day 3 lactate |

GCS, Glasgow Coma Score; ISS, injury severity score; MAP, mean arterial pressure; CPK, creatine phosphokinase; GFR, glomerular filtration rate; COPD, chronic obstructive pulmonary disease; CPB, cardiopulmonary bypass; ICU, intensive care unit; IAB, intra-aortic balloon; WBC, white blood cell count

**Figure S1.** Funnel plot of the studies comparing the patients with and without mechanical ventilation on the occurrence of acute kidney injury.

SE, standard error; OR, odds ratio.

**Figure S2.** Funnel plot of the studies reporting on mechanical ventilation as a risk factor for acute kidney injury in multivariate analysis.

OR, odds ratio; SE, standard error.

**Figure S3.** Funnel plot of the studies comparing low and high positive end-expiratory pressure on occurrence of acute kidney injury.

OR, odds ratio; SE, standard error.

The data reported in the study by Ranieri *et al.* falls outside the plot, marked by the red arrow.

**Figure S4.** Funnel plot of the studies comparing low and high tidal volume ventilation on occurrence of acute kidney injury.

OR, odds ratio; SE, standard error.

The data reported in the study by Ranieri *et al.* falls outside the plot, marked by the red arrow.

**Figure S5.** Forest plot of the studies in which mechanical ventilation clearly preceded acute kidney injury.


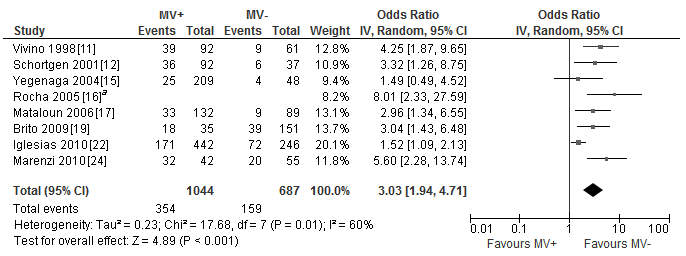


MV+, with mechanical ventilation; MV-, without mechanical ventilation; IV, inverse variance; CI, confidence interval. *^a^* Only OR reported.

**Figure S6.** Forest plot of the studies in which mechanical ventilation did not clearly precede acute kidney injury.


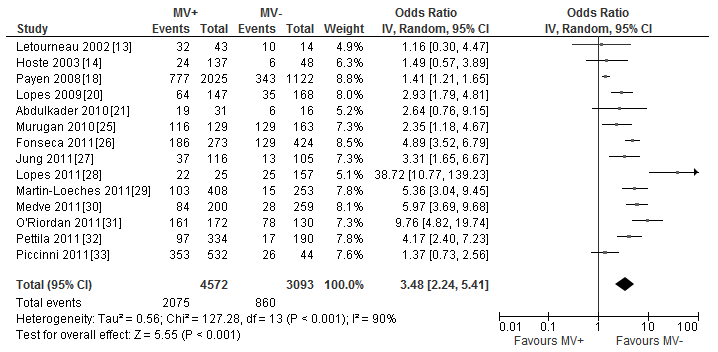


MV+, with mechanical ventilation; MV-, without mechanical ventilation; IV, inverse variance; CI, confidence interval.

**Figure S7.** Forest plot of the studies comparing the patients with and without mechanical ventilation categorised in subgroups whether ALI/ARDS was present or not.

**
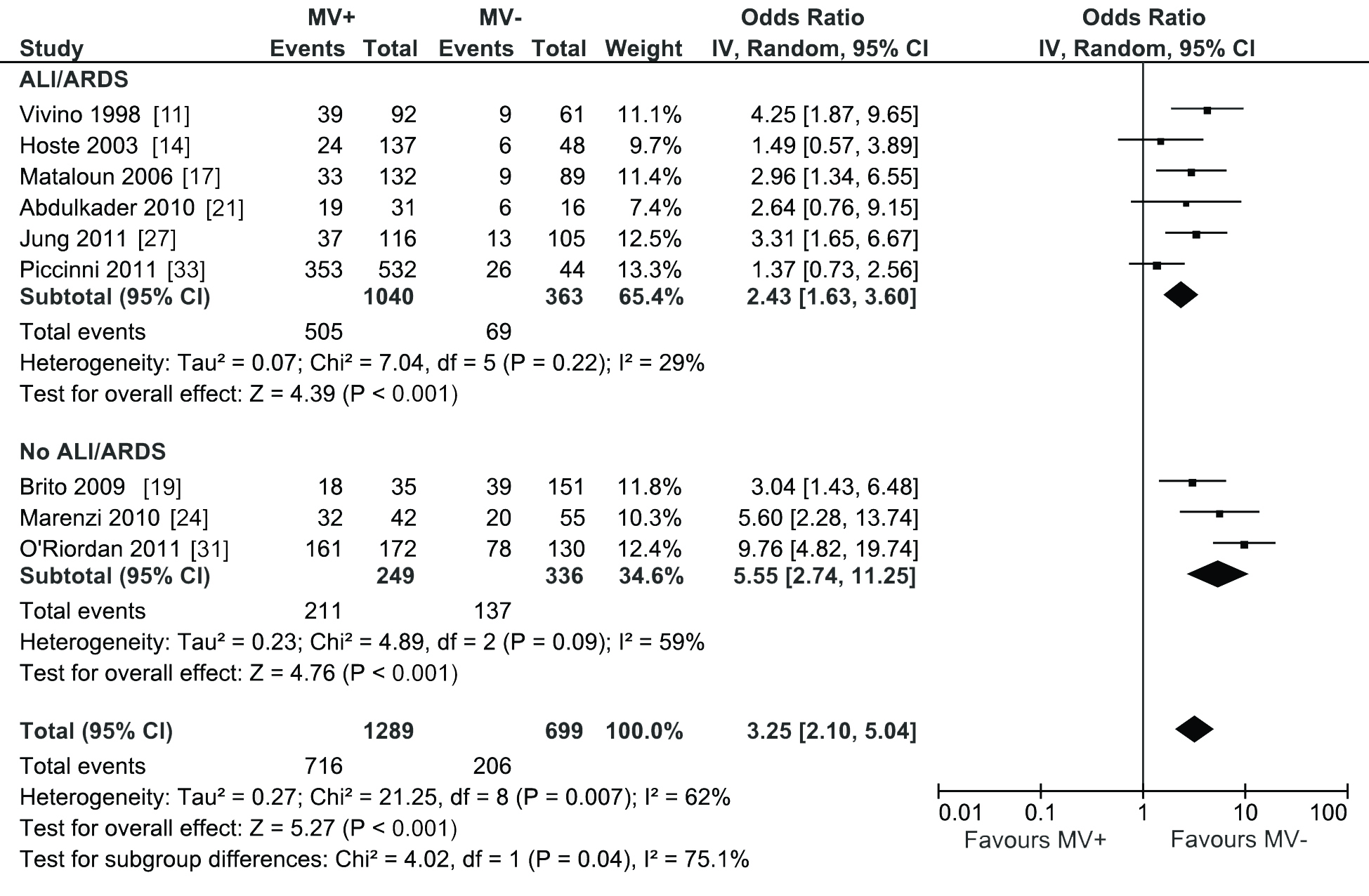
**

MV+, with mechanical ventilation; MV-, without mechanical ventilation; IV, inverse variance; CI, confidence interval.

**Figure S8.** Forest plot comparing non-invasive with invasive mechanical ventilation on the occurrence of acute kidney injury.


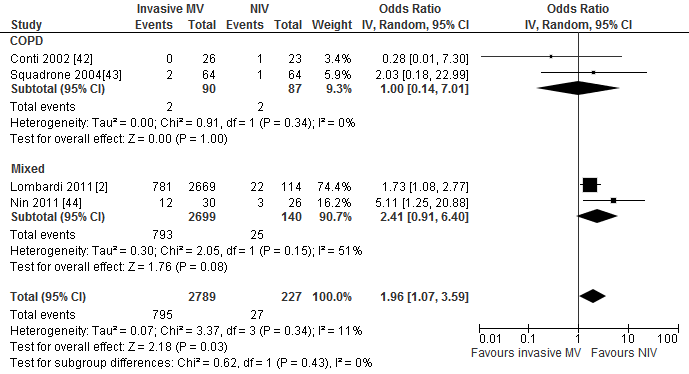


MV, mechanical ventilation; NIV, non-invasive mechanical ventilation; IV, inverse variance; CI, confidence interval; COPD, chronic obstructive pulmonary disease.
